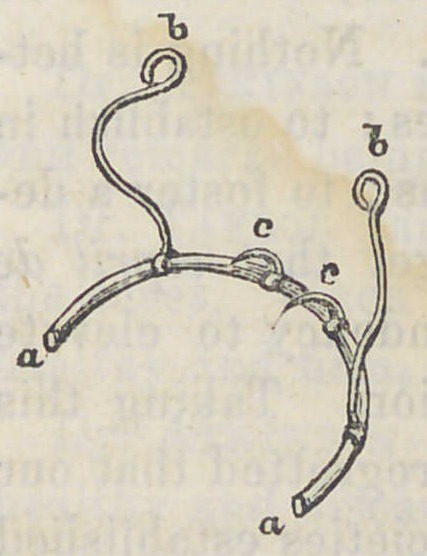# A New Napkin Holder

**Published:** 1861-04

**Authors:** WM. A. Pease


### A NEW NAPKIN HOLDER.

BY WM. A. PEASE.

A LITTLE thing, like a napkin holder, that is convenient and easily adjusted, is often of great service to a dentist. I have many times thanked Dr. Hawes for his, and wondered how I got along before without it. The one that I now offer to the profession I have found very convenient; it is simple, easily adjusted, and not expensive; any dentist can make it, although it will generally be found cheapest to buy it, or at least the material of which it is made, at a dental depot. In connection with Dr. Hawes', it gives a dentist the use of both hands, and such a control of the secretions of the mouth as to enable him to make much better fillings. Dr. Hawes' controls the ducts of Wharton, while this is equally efficient in controlling the secretions of those of Steno; besides, it can be so adjusted, when desirable, as to press the lips and cheeks away from the teeth. Another advantage it possesses consists in a flexible and an adjustable arm (*b*), that can be slipped on at pleasure, which extends up to the duct of Steno, and holds a small napkin steadily against it. Other arms can easily be added to it to meet any emergency or to suit the taste of the operator, altho' I think these are all that will generally be found desirable. Its principal advantage consists in holding a napkin so as to prevent flooding the lower teeth with the secretion of the parotid gland. When used in connection with Dr. Hawes', this should be placed in position first, then his should be adjusted and secured, after which the tooth can be plugged at leisure. A glance at the diagram will show how it is applied—*a a* represent the body of the instrument; *b b* are sliding arms that can be moved to any desirable position, and then the sharp and thin points, *c c*, can be slipped in between the teeth, which is all that is necessary. When the arm, *b*, is used, a small napkin can be wound around the end, which is bent for that purpose.

## Figures and Tables

**Figure f1:**